# Supplementary material for: High-throughput neutralization measurements correlate strongly with evolutionary success of human influenza strains
Source: bioRxiv. 2025 Dec 2:2025.03.04.641544. Originally published 2025 Mar 12. Preprint. [Version 3] doi: 10.1101/2025.03.04.641544 (PMC11952370; doi:10.1101/2025.03.04.641544)
Supplement: Supplement 1 [file NIHPP2025.03.04.641544v3-supplement-1.pdf]

bioRxiv preprint doi: https://doi.org/10.1101/2025.03.04.641544; this version posted December 2, 2025. The copyright holder for this preprint (which was not certified by peer review) is the author/funder, who has granted bioRxiv a license to display the preprint in perpetuity. It is made available under aCC-BY 4.0 International license.

Supplemental tables

Northern Hemisphere vaccine season	Cell-passaged vaccine strain	Egg-passaged vaccine strain	Cell-passaged vaccine strain GenBank	Cell-passaged vaccine strain GISAID	Egg-passaged vaccine strain GenBank	Egg-passaged vaccine strain GISAID	Amino-acid mutations in egg-passaged strain relative to cell-passaged strain
2014-2015	A/Texas/50/2012	A/Texas/50/2012X-223A	AFH34947.1	EPI_ISL_122006		EPI_ISL_138982	G186V, S219V, I226N
2015-2016	A/Switzerland/9715293/2013	A/Switzerland/9715293/2013NIB-88	QBM69707.1	EPI_ISL_166310	APF46463.1	EPI_ISL_198223	I140R, G186V, S219Y
2016-2018	A/HongKong/4801/2014	A/HongKong/4801/2014egg	AJM70923.1	EPI_ISL_165554	WCF71375.1	EPI_ISL_176512	N96S, T160K, L194P
2018-2019	A/Singapore/INFIMH-16-0019/2016	A/Singapore/INFIMH-16-0019/2016X-307A	QQY97257.1	EPI_ISL_225834	WCF71352.1	EPI_ISL_293527	T160K, L194P, D225G
2019-2020	A/Kansas/14/2017	***A/Kansas/14/2017X-327 (strain not included)***	QIC35448.1	EPI_ISL_292575	WMZ80193.1	EPI_ISL_346457	G186V, D190N, S219Y
2020-2021	A/HongKong/45/2019	A/HongKong/2671/2019	WMW30913.1	EPI_ISL_347938	WMW30924.1	EPI_ISL_400888	T160I, G186V, D225N, N312S
2021-2022	A/Cambodia/e0826360/2020	A/Cambodia/e0826360/2020egg		EPI_ISL_944639		EPI_ISL_806547	T160K, S186R
2022-2024	A/Darwin/6/2021	A/Darwin/9/2021	UJS43281.1	EPI_ISL_3534319	WEY08928.1	EPI_ISL_2233240	G53D, D186N, D225G
2024-2025	A/Massachusetts/18/2022	A/Thailand/8/2022	WIG19387.1	EPI_ISL_13897304	XLJ76603.1	EPI_ISL_16014504	V182I, F195Y

Supplemental table 1. Cell-passaged and egg-passaged H3N2 strains chosen for the seasonal human influenza vaccine each season 2014-2024.

For each Northern Hemisphere seasonal influenza vaccine from 2014-2024, we list the cell- and egg-based vaccine strains and the GenBank and GISAID identification numbers linked to each sequence. We also note the amino-acid mutations present in each egg-passaged strain relative to its cell-passaged counterpart from the same vaccine season. Note the egg-passaged virus for the 2019-2020 season is listed (A/Kansas/14/2017X-327), but this strain did not grow to sufficiently high titers to be compatible with our assay and was excluded from the library.

bioRxiv preprint doi: https://doi.org/10.1101/2025.03.04.641544; this version posted December 2, 2025. The copyright holder for this preprint (which was not certified by peer review) is the author/funder, who has granted bioRxiv a license to display the preprint in perpetuity. It is made available under aCC-BY 4.0 International license.

Supplemental figures

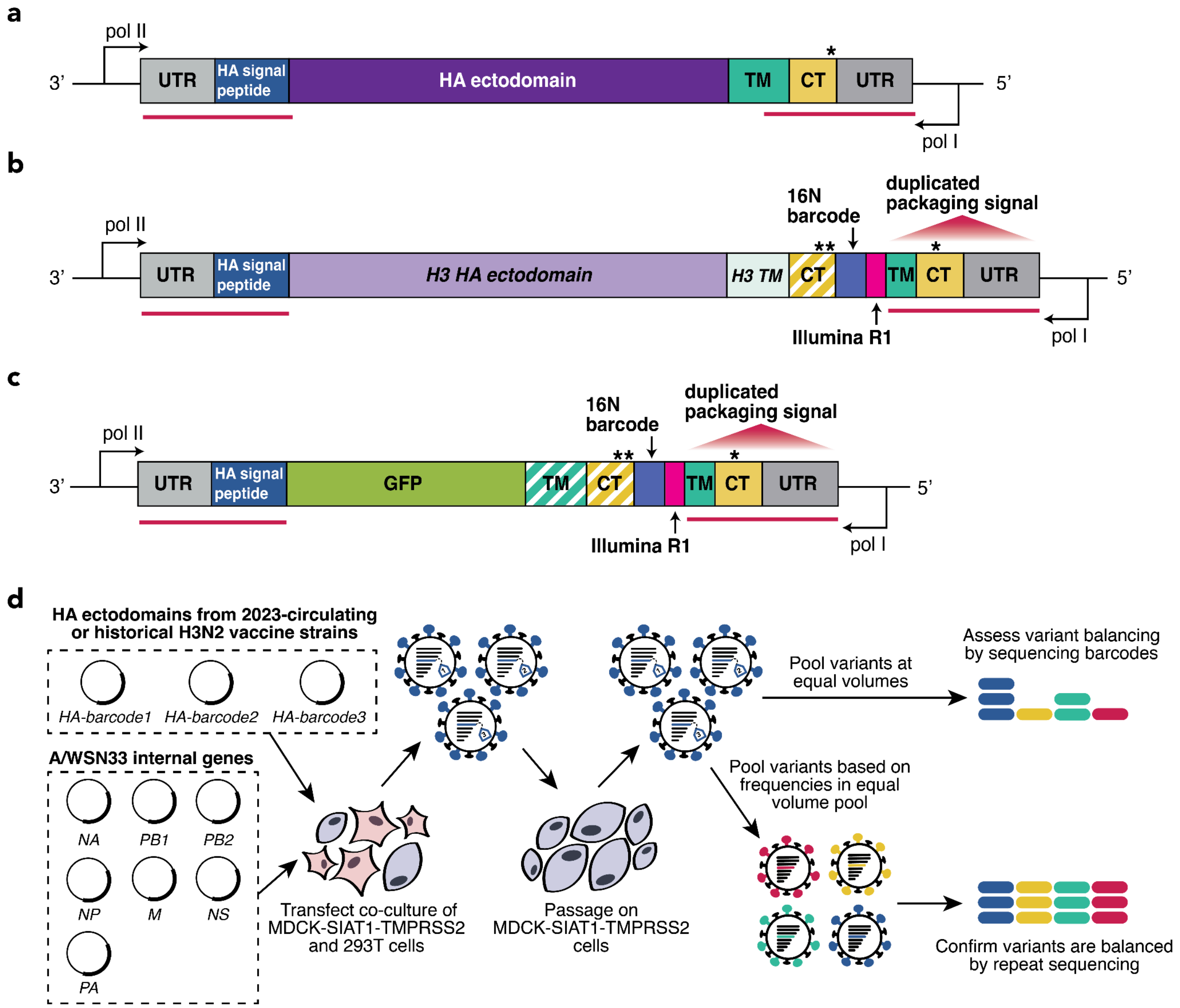

Supplemental figure 1. Design of chimeric barcoded HA construct and virus library generation.

All HA gene segments in panels **a-c** are shown so that the encoded HA protein is oriented N-terminal to C-terminal, which is the 3′ to 5′ orientation of the negative-sense influenza viral genome. Stop codons are indicated with asterisks. **(a)** A schematic of the unmodified A/WSN/1993(H1N1) HA gene segment and partial upstream and downstream segments of the bidirectional influenza reverse-genetics plasmid[97]. The segment-specific regions of vRNA required for proper packaging of genome segments into nascent virions, or "packaging signals," are denoted as red lines spanning both the untranslated regions (UTRs) and HA coding sequence, which includes the signal peptide, ectodomain, transmembrane (TM) and C-terminal (CT) regions. Outside of the HA vRNA-encoding regions, black lines show the upstream pol II promoter (for transcription of viral mRNA) and the downstream pol I promoter (for

bioRxiv preprint doi: https://doi.org/10.1101/2025.03.04.641544; this version posted December 2, 2025. The copyright holder for this preprint (which was not certified by peer review) is the author/funder, who has granted bioRxiv a license to display the preprint in perpetuity. It is made available under aCC-BY 4.0 International license.

transcription of negative sense vRNA to be packaged in virus particles) that are found in the bidirectional influenza reverse genetics plasmid[97]; note these are not part of the actual viral RNAs. **(b)** A schematic of the chimeric H3 HA barcoded construct, similar to those in Loes et al.[43] and Welsh et al.[29] Where the HA gene coding sequence exactly match A/WSN/1993(H1N1) (at the upstream HA signal peptide and in most of the duplicated packaging signal) are colored as in panel **a**. The remainder of the downstream portions of the construct have changed, and are re-colored accordingly. The HA ectodomain is replaced with H3 library HA ectodomain sequences. The downstream TM domain is fixed to an H3 HA consensus sequence. The CT matches the A/WSN/1993(H1N1) protein sequence. Both the H3 TM and the A/WSN/1993(H1N1) CT are synonymously recoded to avoid complementation with the downstream duplicated packaging signal. The stop codon at the end of the HA coding region is duplicated to prevent polymerase read-through. A 16-nucleotide barcode segment follows these dual HA gene stop codons, followed by the Illumina R1 sequence necessary for preparing barcoded amplicons for sequencing. Finally, to preserve proper packaging of the entire gene segment, there is a duplicated packaging signal from A/WSN/1993(H1N1), including the partial TM, CT and downstream UTR regions. As in the constructs described in Loes et al.[43], an additional stop codon is engineered that will be in-frame if this duplicated packaging signal replaces the partial packaging signal in the HA coding region; therefore, any HA gene segments that lose the barcoded segment should generate a truncated, non-functional HA protein. **(c)** A schematic of the construct from which the barcoded RNA spike-in are generated, identical to that in Loes et al. This construct is designed to produce barcoded RNA molecules identical to the barcoded HA segment described in panel **b**, but with a GFP sequence in place of the HA ectodomain sequence. As in **b**, the TM and CT domain are from A/WSN/1993(H1N1), and are synonymously recoded in the same manner to provide an identical priming region as incorporated in the HA construct. **(d)** A schematic of viral library generation, identical to that described in Loes et al.[43] To express each HA variant on the surface of influenza virus particles, we use a reverse genetics protocol[97] in which influenza virus gene segments are each expressed on separate plasmids and transfected into a co-culture of 293T cells and Madin-Darby Canine Kidney (MDCK) cells overexpressing 2,6-sialyltransferase (SIAT1) and transmembrane protease serine 2 (TMPRSS2). The 3 sequence-confirmed barcoded HA variants per HA sequence are pooled at this stage, and all other non-HA segments are from the lab-adapted H1N1 strain A/WSN/1993. Any changes in neutralization potency can therefore be attributed to an antigenic change within HA. Each per-strain pool of HA barcoded viruses is then passaged on MDCK-SIAT1-TMPRSS2 cells to reduce plasmid carry-over and improve viral titers. After this step, the barcoded HA strains are pooled to create a barcoded HA variant library, which can then be sequenced to assess HA strain balancing. Then, using this information to more equally represent strains, the final, balanced pool is generated.

bioRxiv preprint doi: https://doi.org/10.1101/2025.03.04.641544; this version posted December 2, 2025. The copyright holder for this preprint (which was not certified by peer review) is the author/funder, who has granted bioRxiv a license to display the preprint in perpetuity. It is made available under aCC-BY 4.0 International license.

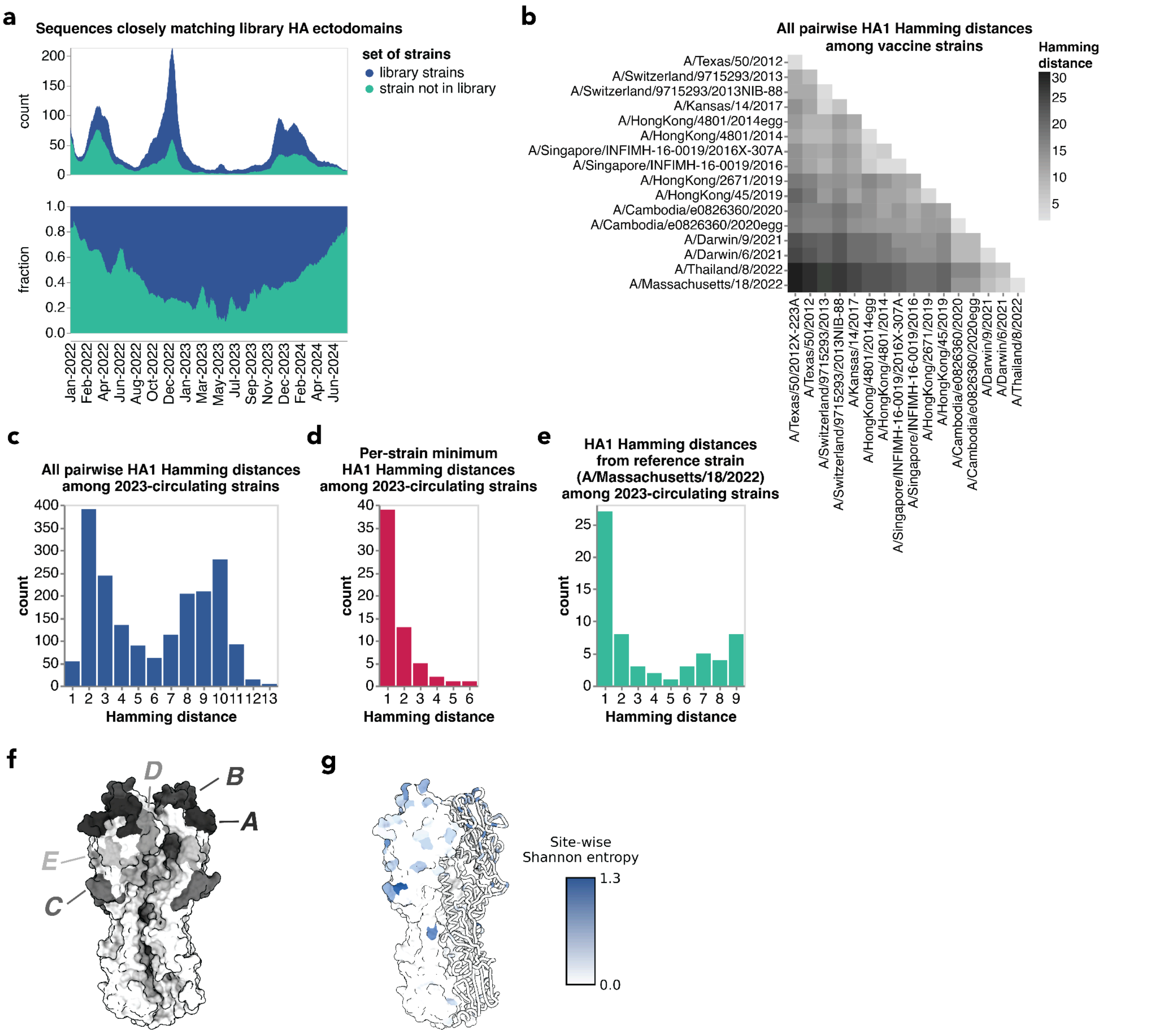

Supplemental figure 2. HA diversity among the strains in the library.

(a) Count and fraction of all sequenced human H3N2 with HAs that closely match HA ectodomain protein sequences in our library. A close match is defined as being within one amino acid mutation in the HA ectodomain. This plot differs from **Figure 2a** by showing matches for the full HA ectodomain rather than just HA1. **(b)** Heatmap of pairwise HA1 amino acid sequence Hamming distances between all vaccine strains in the library. **(c)** Distribution of pairwise HA1 amino acid sequence Hamming distances between all 2023-circulating strains in the library. **(d)** The shortest HA1 amino acid sequence Hamming distance between each 2023-circulating strain in the library and another 2023-circulating strain. **(e)** HA1 amino acid sequence Hamming distance between each 2023-circulating strain in the library and the cell-passaged H3 component of the 2024–2025 seasonal influenza vaccine (A/Massachusetts/18/2022). **(f)** H3 HA trimer with antigenic regions (as defined by Munoz and Deem[45]) colored and labeled. The H3 HA structure is from A/Victoria/361/2011 (PDB: 4O5N[108]).

bioRxiv preprint doi: https://doi.org/10.1101/2025.03.04.641544; this version posted December 2, 2025. The copyright holder for this preprint (which was not certified by peer review) is the author/funder, who has granted bioRxiv a license to display the preprint in perpetuity. It is made available under aCC-BY 4.0 International license.

(g) Site-wise Shannon entropy calculated from HA ectodomain sequences from all 2023-circulating strains and vaccine strains, mapped onto the same H3 HA trimer as in **f**. Most high-entropy sites fall within previously defined antigenic regions.

bioRxiv preprint doi: https://doi.org/10.1101/2025.03.04.641544; this version posted December 2, 2025. The copyright holder for this preprint (which was not certified by peer review) is the author/funder, who has granted bioRxiv a license to display the preprint in perpetuity. It is made available under aCC-BY 4.0 International license.

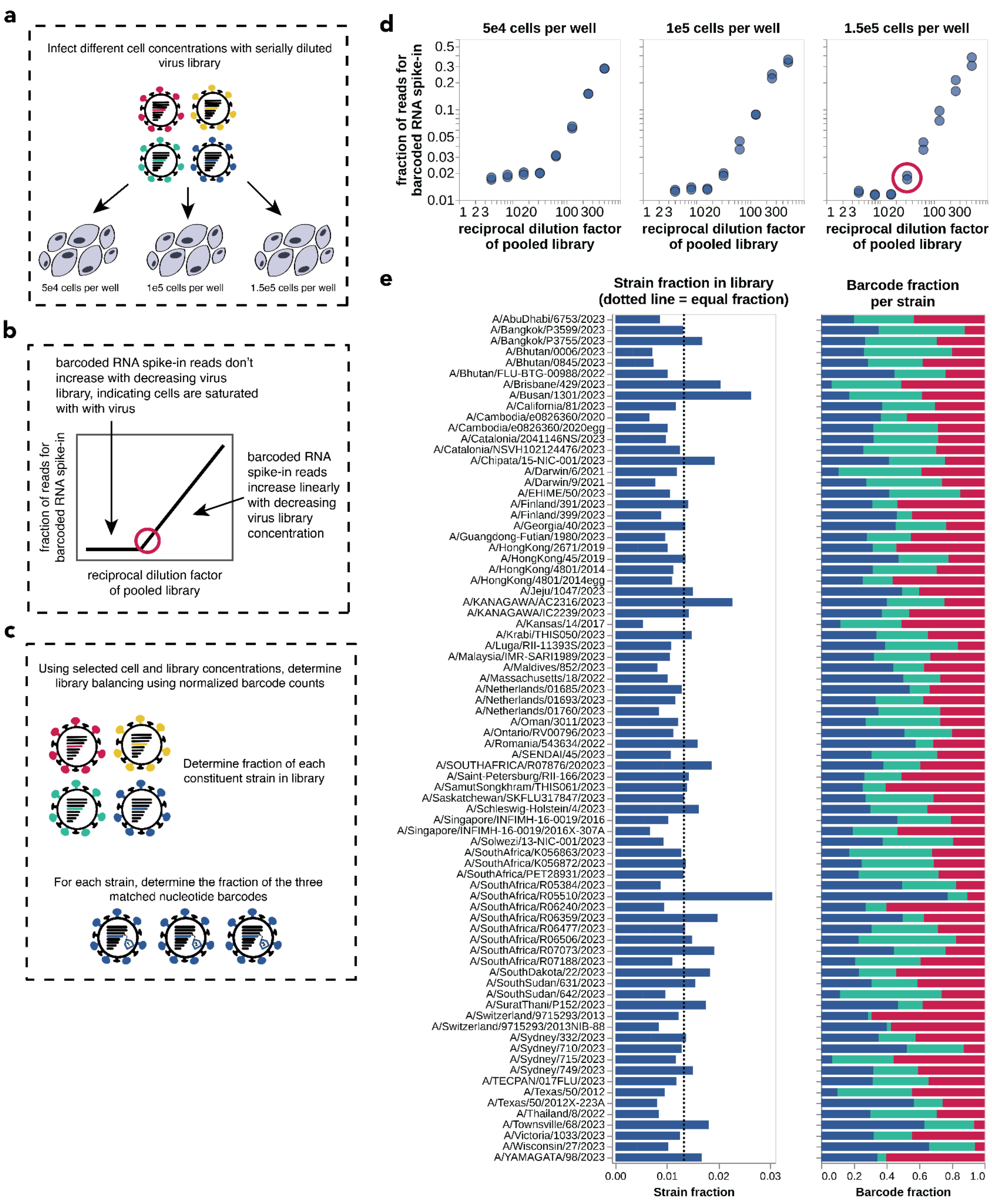

Supplemental figure 3. Determining and validating the cell density and viral library concentration for sequencing-based neutralization assays.

(a) To optimize the cell density and virus library dilution for the sequencing-based neutralization assay, we infected varying numbers of cells with serial dilutions of the virus

bioRxiv preprint doi: https://doi.org/10.1101/2025.03.04.641544; this version posted December 2, 2025. The copyright holder for this preprint (which was not certified by peer review) is the author/funder, who has granted bioRxiv a license to display the preprint in perpetuity. It is made available under aCC-BY 4.0 International license.

library. While Loes et al.[43] used 5e4 cells per well, the assay's performance at higher cell concentrations was unknown. During the lysis step, we spiked in barcoded RNAs (as shown in **Figure 1**) to quantify the read counts at each virus library dilution across the tested cell concentrations. **(b)** The optimal conditions (indicated by the red circle) are those that maximize the number of virus particles added to each well while remaining in the linear range where transcriptional output scales linearly with the amount of infectious virus. By maximizing the number of infectious virus particles added to each well we reduce statistical noise due to bottlenecking of the library. Remaining in the linear range is crucial for several reasons. First, when cells are saturated with infectious virus, changes in an increase in the number of virions infecting a cell does not lead to a linearly proportional increase in vRNA transcription, which is the critical readout for this assay. Second, selecting a dilution at the beginning of the linear range provides the largest window for detecting decreases in viral barcodes (i.e., virus neutralization). **(c)** Using each cell density and virus dilution from the experiment in **a**, we determine the fraction of each strain in the library and the relative fraction for each barcode per strain. This analysis determines if the library is reasonably equally-balanced at the chosen experimental conditions. **(d)** The fraction of reads corresponding to barcoded RNA spike-in is plotted against different virus dilutions for per-well cell counts of 5e4, 1e5, and 1.5e5 cells. Each point represents a replicate serial dilution of the virus library. As cell concentration increases, the fraction of reads for barcoded RNA spike-in at higher virus library concentrations decreases, demonstrating the library MOI decreases (at the same library dilution factors) with increasing cell concentration. This trend is expected, as the number of infectious particles remains constant at each dilution, while the number of infectable cells increases. **(e)** At the chosen cell density and library concentration (1.5e5 cells per well and a 1:32 dilution of the virus library), we show the calculated strain fraction in the library (left) and the fraction of each barcode per strain (right) after library balancing and re-pooling (**Supplemental Figure 1d**). Each row of the barplots represents a different strain. In the strain fraction panel (left), the dotted line indicates the ideal fraction where all strains would be equally represented. In the barcode fraction per strain panel (right), each color represents a different barcode, and the width of each stacked bar indicates the fraction of reads attributed to that barcode for each strain.

bioRxiv preprint doi: https://doi.org/10.1101/2025.03.04.641544; this version posted December 2, 2025. The copyright holder for this preprint (which was not certified by peer review) is the author/funder, who has granted bioRxiv a license to display the preprint in perpetuity. It is made available under aCC-BY 4.0 International license.

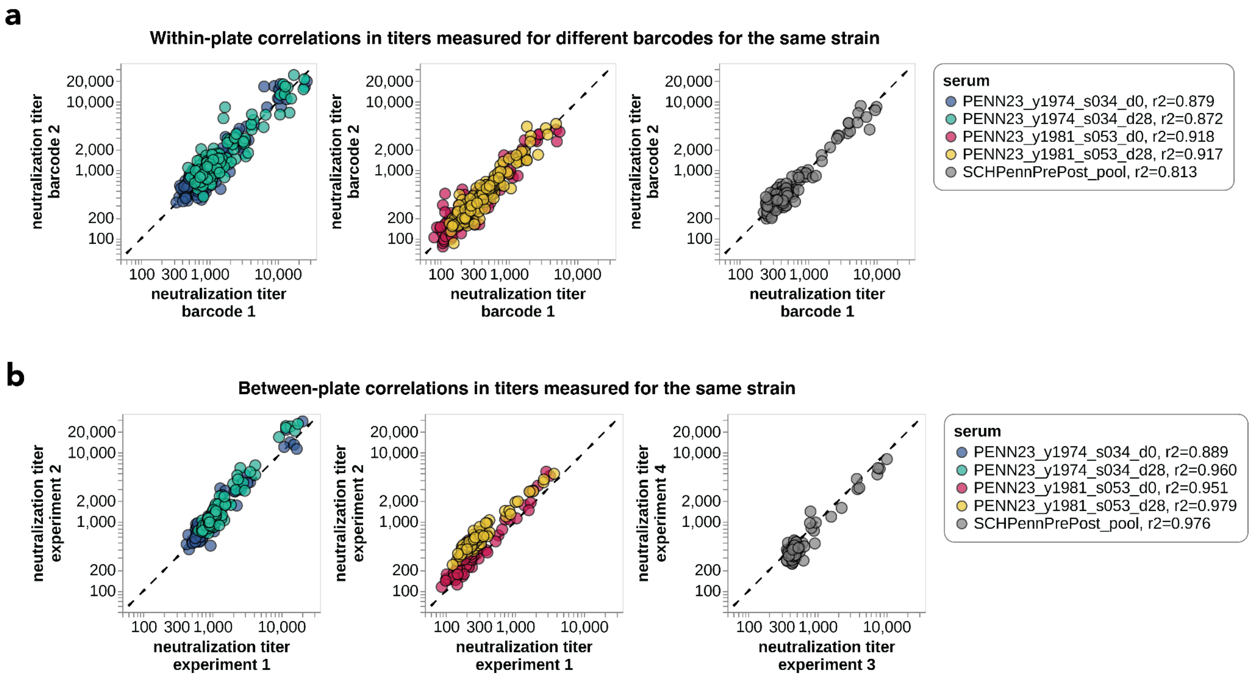

Supplemental figure 4. Within- and between-plate titer measurements are highly correlated.

(a) The correlation between different barcodes for the same HA measured for a subset of individual sera and a serum pool. Each point represents the neutralization titers measured for two different barcodes for a single virus on the same plate. **(b)** Correlations between neutralization titers measured in separate experiments performed on separate days. Each point represents the neutralization titer for a single virus-serum pair (i.e., the median of the three replicate barcodes for that virus on each plate) as measured in two different experiments completed and sequenced on different days.

bioRxiv preprint doi: https://doi.org/10.1101/2025.03.04.641544; this version posted December 2, 2025. The copyright holder for this preprint (which was not certified by peer review) is the author/funder, who has granted bioRxiv a license to display the preprint in perpetuity. It is made available under aCC-BY 4.0 International license.

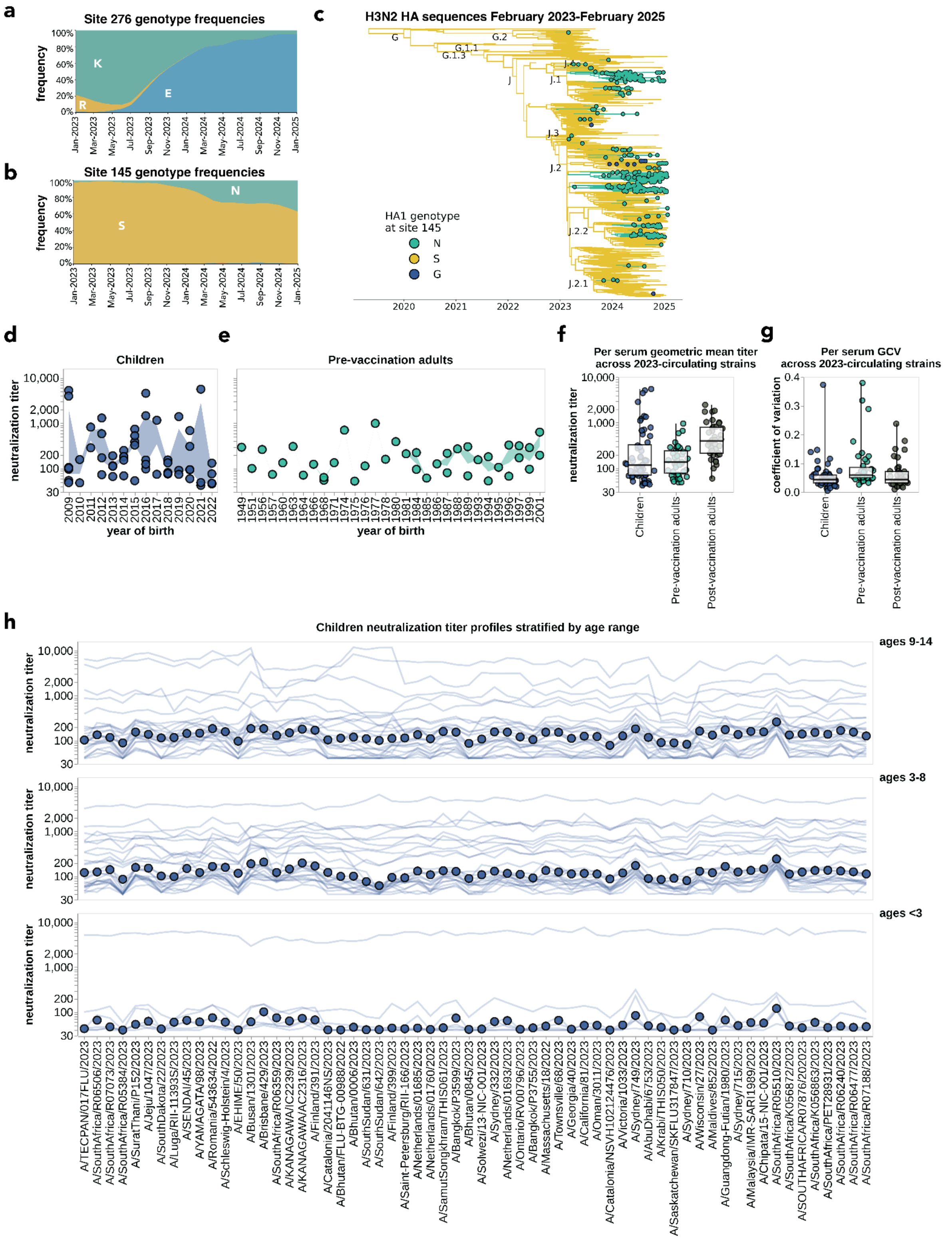

Supplemental figure 5. H3 HA phylodynamics, birth year cohorts and age cohorts explain some patterns in neutralization titers.

bioRxiv preprint doi: https://doi.org/10.1101/2025.03.04.641544; this version posted December 2, 2025. The copyright holder for this preprint (which was not certified by peer review) is the author/funder, who has granted bioRxiv a license to display the preprint in perpetuity. It is made available under aCC-BY 4.0 International license.

(a) Frequency of different amino-acid mutations at site 276 among human H3N2 HA sequences over time. Notably, a 276E mutation on the background of 140K (the J subclade-defining mutation) came to define the J.2 subclade. The J.2 subclade predominated during the 2023-2024 season. **(b)** Frequency of different amino-acid mutations at site 145 among human H3N2 HA sequences over time. **(c)** Phylogenetic tree of H3N2 HA sequences, where branches are colored based on their amino acid identity at site 145. Nextstrain-defined subclades are labeled at nodes. **(d)** For each serum sample in the children's cohort, the geometric mean neutralization titer across 2023-circulating strains was calculated and plotted by birth year. The shaded regions show the interquartile range (IQR) of neutralization titers for each birth year group. **(e)** The same analysis described in **d** was performed for the pre-vaccination adult cohort. **(f)** The distribution of geometric mean neutralization titers taken over all 2023-circulating strains for all sera in each cohort. Each point represents the geometric mean neutralization titer of a single serum against 2023-circulating strains. **(g)** The distribution of the geometric coefficient of variation (GCV) over all 2023-circulating strains for all sera in each cohort. Each point represents the geometric coefficient of variation of a single serum across 2023-circulating strains. **(h)** Neutralization titer profiles across all individuals from the children cohort, re-plotting the data from **Figure 3b**, but now stratified by age groups.

bioRxiv preprint doi: https://doi.org/10.1101/2025.03.04.641544; this version posted December 2, 2025. The copyright holder for this preprint (which was not certified by peer review) is the author/funder, who has granted bioRxiv a license to display the preprint in perpetuity. It is made available under aCC-BY 4.0 International license.

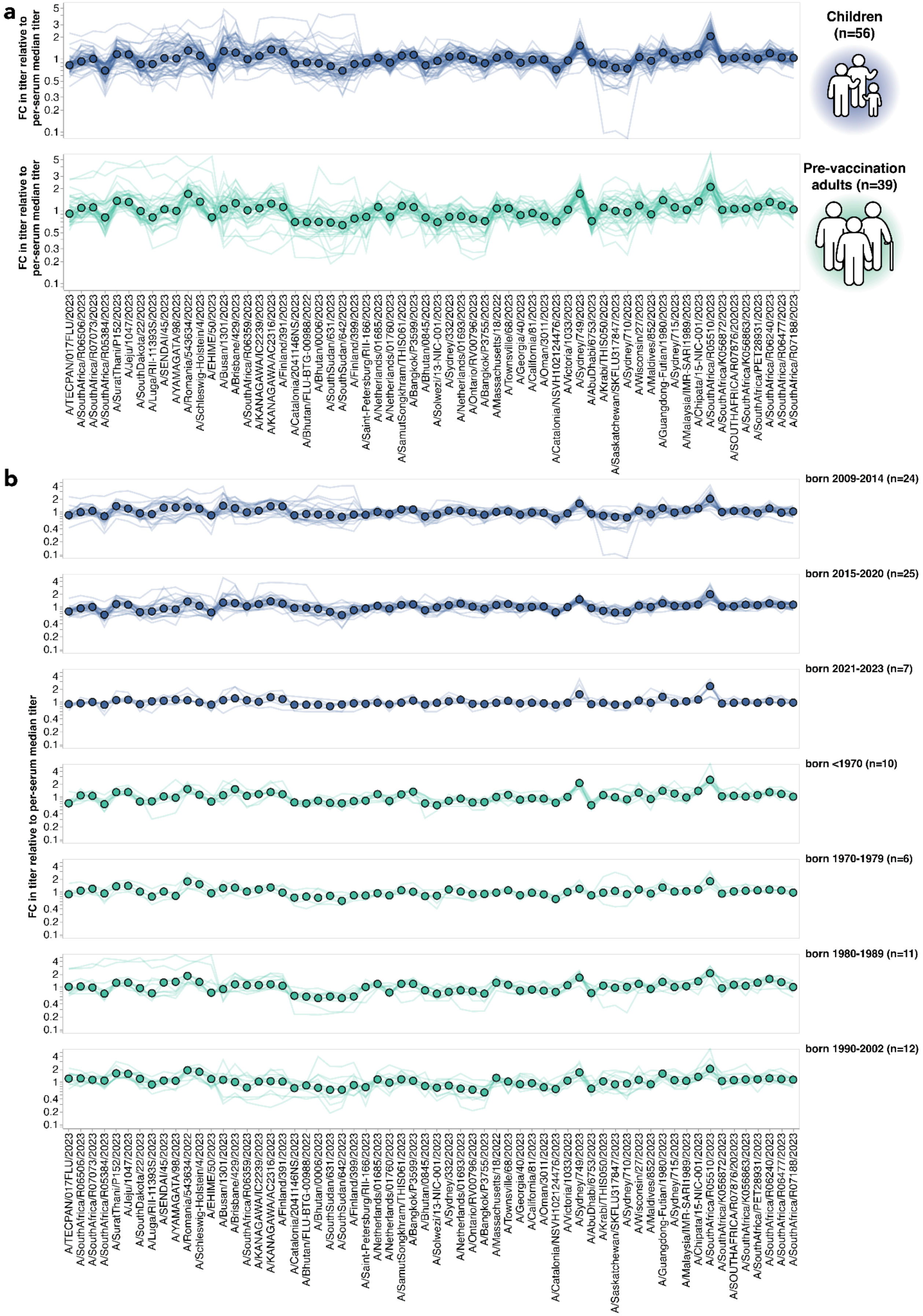

bioRxiv preprint doi: https://doi.org/10.1101/2025.03.04.641544; this version posted December 2, 2025. The copyright holder for this preprint (which was not certified by peer review) is the author/funder, who has granted bioRxiv a license to display the preprint in perpetuity. It is made available under aCC-BY 4.0 International license.

Supplemental figure 6. Fold change in titer for each viral strain relative to the median titer across all strains for each children and pre-vaccination adult sera.

(a) For each serum, we determined the median titer of that serum across all recent viral strains, and then plotted the fold change relative to that median for each serum against each virus. Each thin line represents the fold changes relative to the median for a single serum, and the circles represent the median of the fold changes for each virus across sera. **(b)** The same fold-changes in titer relative to the median as in **(a)**, faceted by more fine-grained age groups.

bioRxiv preprint doi: https://doi.org/10.1101/2025.03.04.641544; this version posted December 2, 2025. The copyright holder for this preprint (which was not certified by peer review) is the author/funder, who has granted bioRxiv a license to display the preprint in perpetuity. It is made available under aCC-BY 4.0 International license.

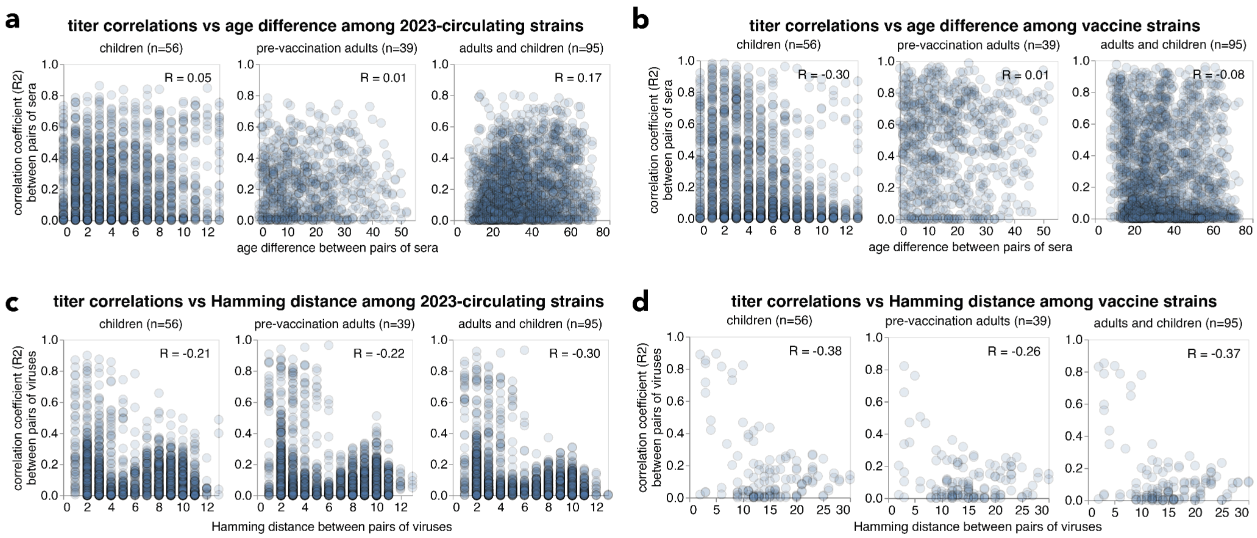

Supplemental figure 7. Neutralizing titer correlations and their relationship with age difference between pairs of sera and Hamming distance between pairs of viruses

The correlation between titers from pairs of sera was calculated across titers for all 2023-circulating strains **(a)** and vaccine strains **(b)** and then correlated with the age difference in years between each pair of sera. Similarly, the correlation between neutralizing titers across all sera to pairs of all 2023-circulating strains **(c)** and vaccine strains **(d)** were compared to the amino acid-level Hamming distances between pairs of viruses.

bioRxiv preprint doi: https://doi.org/10.1101/2025.03.04.641544; this version posted December 2, 2025. The copyright holder for this preprint (which was not certified by peer review) is the author/funder, who has granted bioRxiv a license to display the preprint in perpetuity. It is made available under aCC-BY 4.0 International license.

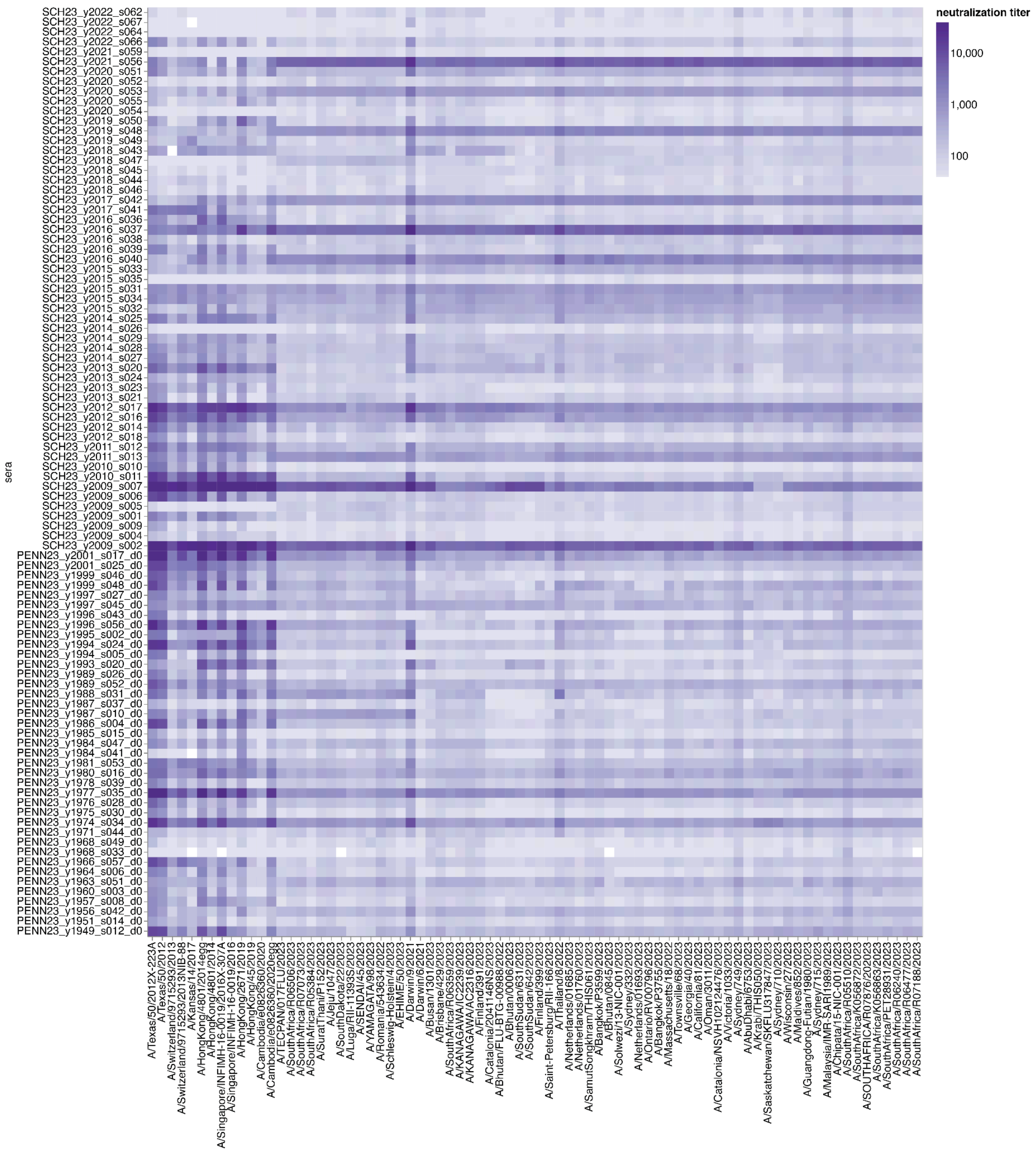

Supplemental figure 8. Neutralizing titers across all vaccine and 2023-circulating strains plotted as a heatmap

All children and pre-vaccination adults are shown in increasing age order on the y-axis, and the neutralizing titer against each strain in the library is indicated on a log color scale. This figure shows the same data plotted in Figure 3 in a different way.

bioRxiv preprint doi: https://doi.org/10.1101/2025.03.04.641544; this version posted December 2, 2025. The copyright holder for this preprint (which was not certified by peer review) is the author/funder, who has granted bioRxiv a license to display the preprint in perpetuity. It is made available under aCC-BY 4.0 International license.

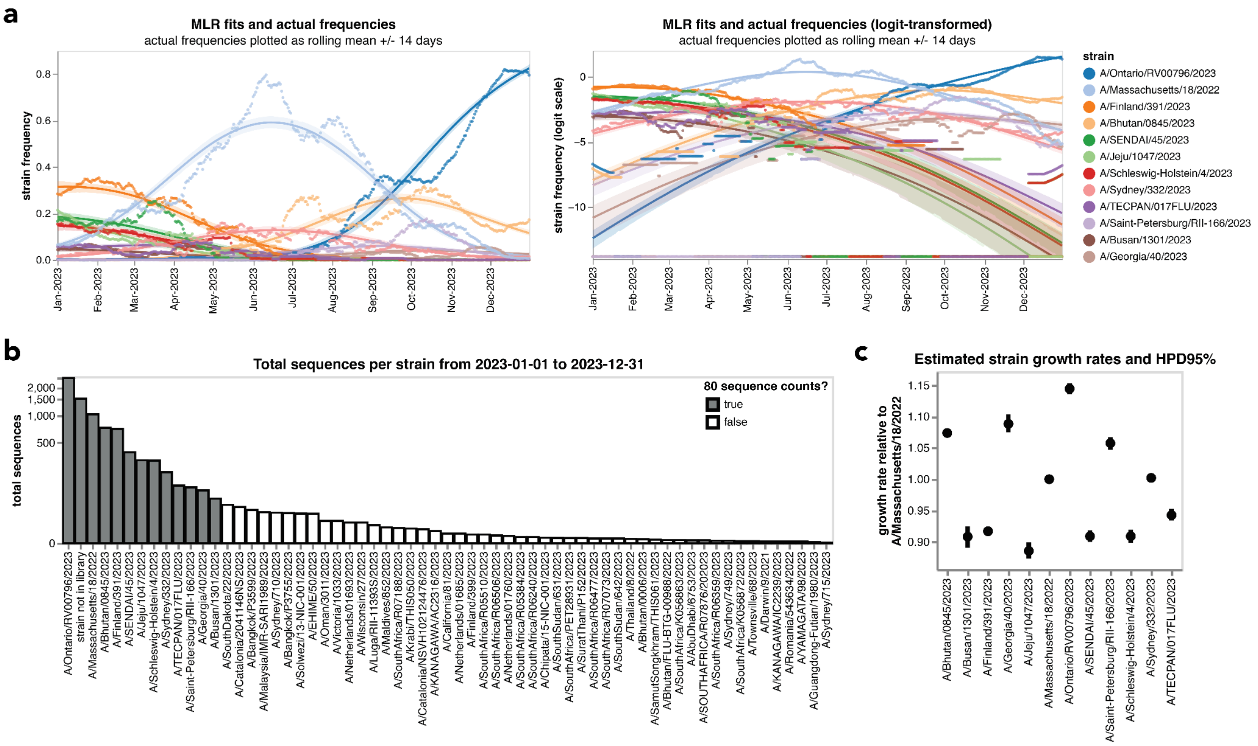

Supplemental figure 9. Multinomial logistic regression model fits of strain growth rates.

(a) The multinomial logistic regression model fits overlaid on sequence frequencies as in **Figure 5a** (left), in comparison to the logit-transformed sequence frequencies (right). The multinomial logistic regression implementation defines two parameters per variant: the initial frequency and the growth rate. The absolute variant growth rate is calculated from the slope of these logit-transformed variant frequencies. We can use these absolute growth rates to estimate each variant's relative growth rate (i.e., their relative fitness) by normalizing that variant's growth rate relative to a predefined baseline variant (elsewhere, that baseline variant is A/Massachusetts/18/2022). **(b)** The total number of H3N2 influenza sequences collected in 2023 that closely matched each library strain HA1 sequence. In order to make a growth estimate by multinomial logistic regression, we set the threshold of at least 80 sequence counts, where each sequence needed to be an exact match or within one amino-acid mutation of a given library sequence. The reason for this threshold is that it is only possible to estimate growth rates if there are enough sequence counts to reliably determine the frequency trajectory of the strain. Twelve strains met this threshold. **(c)** The estimated relative growth rates from multinomial logistic regression and their 95% highest posterior density intervals (HPD95%) relative to a baseline strain (A/Massachusetts/18/2022).

bioRxiv preprint doi: https://doi.org/10.1101/2025.03.04.641544; this version posted December 2, 2025. The copyright holder for this preprint (which was not certified by peer review) is the author/funder, who has granted bioRxiv a license to display the preprint in perpetuity. It is made available under aCC-BY 4.0 International license.

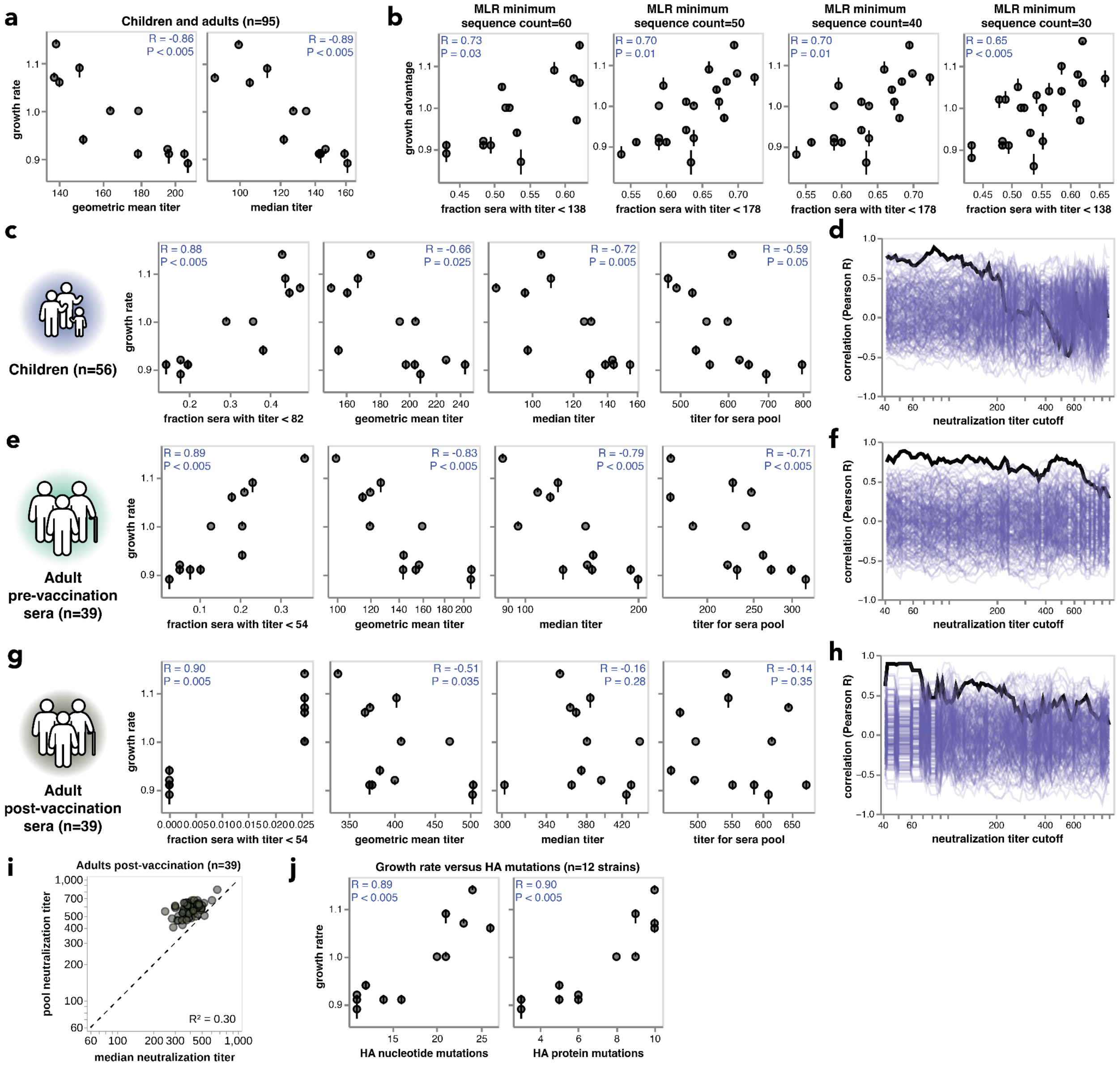

Supplemental figure 10. Additional growth rate comparisons with neutralization titers and evolutionary distances.

(a) Correlations between estimated growth rates for the 12 strains and the per-strain median and geometric mean neutralization titers across 95 children and adults. **(b)** Correlations between the fraction of individuals with low neutralization titers across 95 children and adults and the strain growth rates estimated with a range of cutoffs for how many sequencing counts a strain must have to estimate its growth rate. **(c)** These plots are comparable to those in **Figure 5c** and panel **a** of this figure, but using only the 56 children sera. **(d)** This plot is identical to that in **Figure 5d** except it uses titer data for only the 56 children sera. **(e,f)** The same correlations and analysis as described in **c,d**, but from titers measured from the 39 pre-vaccination adult sera. **(g,h)** The same correlations and analysis as described in **c,d**, but from titers measured from the

bioRxiv preprint doi: https://doi.org/10.1101/2025.03.04.641544; this version posted December 2, 2025. The copyright holder for this preprint (which was not certified by peer review) is the author/funder, who has granted bioRxiv a license to display the preprint in perpetuity. It is made available under aCC-BY 4.0 International license.

39 post-vaccination adult sera. **(i)** Correlations between titers measured from pooled sera and the median of individually-measured titers for post-vaccination adults. Each dot corresponds to the pooled or median titer for a given 2023-circulating library strain. **(j)** Correlations between estimated growth rates for the 12 strains and the number of HA ectodomain nucleotide mutations (left) and HA ectodomain amino acid mutations (right).
